# Observing the Unobservable: Migrant Selectivity and Agentic Individuality Among Higher Education Students in China and Europe

**DOI:** 10.3389/fsoc.2020.00009

**Published:** 2020-03-13

**Authors:** Yasemin Nuhoḡlu Soysal, Héctor Cebolla-Boado

**Affiliations:** ^1^Department of Sociology, University of Essex, Colchester, United Kingdom; ^2^Department of Sociology, National University of Distance Education (UNED), Madrid, Spain

**Keywords:** agentic individual, migrant selectivity, unobservable selectivity, higher education, educational migrants, China, Europe

## Abstract

The research in migrant selectivity largely overlooks the broader institutional processes that shape the extent to which migrants from different backgrounds are indeed positively selected. This is particularly true in the case of highly skilled migrants, whose selection may not be conditioned by migration but by education. This paper deals with this limitation by studying individual characteristics, which are often treated as unobserved selectivity, among a specific flow of educational migrants in Europe, namely, Chinese higher education students. To do so, we use a unique representative multi-country dataset of about 8,000 Chinese international students and their native-born counterparts in China, the UK, and Germany. Our evidence rules out positive selection of migrants on individuality traits such as ambition, creativity, or being a risk-taker or independently minded. This supports our argument that the prevalence of agentic models of individuality is embedded in tertiary education on a global level.

## Introduction

Migration research has historically faced the analytical problem of isolating the effect of migration on specific integration outcomes from that of selection on confounders, which simultaneously create a push for migration and differential outcomes by migrant status (Cebolla-Boado and Soysal Nuhoḡlu, 2017). This line of thinking posits that the causal connection between migration and migrant/native-born differentials could be overstated, since both migration and differentials in selected outcomes are caused by hidden confounders that are generally difficult to uncover in empirical research. Selection is at the heart of this problem. The idea that migrants are a self-selected population, that is, they are not a representative sample of population in origin, has been suggested as one of the explanations for migrant/native-born differentials (Chiswick, 1999) such as migrant educational optimism or better health outcomes.

Research designs in tune with this line of research are complex and require comparing migrants and non-migrants in countries of origin in addition to using native-born citizens in destination as a second reference group. Research into international migration seems to be slowly moving toward this approach, from a destination perspective (based on comparisons between migrants and native-born citizens in destination) to an origin-destination approach that includes comparisons with native-borns in origin and destination (Massey and Zenteno, 2000; Garip, 2016; Guveli et al., 2016; Mussino et al., 2018). Such move helps to control for the inevitable selection bias whereby emigrants are not a representative sample of the population who decided not to migrate. When not incorporating the origin perspective, research confuses the impact of selection with that of conventional variables in migration research such as integration policies in destination. In other words, when dynamics in origin are ignored, it is not possible to fully understand the reasons why migrants and native-born citizens differ in key integration outcomes in destination countries.

Migrants are expected to be selected on both observed and unobserved characteristics. While observable selectivity is increasingly addressed in immigration literature, due to data difficulties, we know less about empirical patterns and theoretical underpinnings of unobservable selectivity. Relevant observable characteristics are widely registered in mainstream surveys using diverse and measurable indicators of social background such as education, social status, income, and family background among others. Research into selection by observable characteristics is thus possible largely using existing datasets (Ichou, 2014; Feliciano and Lanuza, 2016, 2017; van de Werfhorst and Heath, 2018). However, studying selection on unobservables is by definition less straightforward and thus more difficult to translate into empirical analysis. When studied, unobservables are often reduced to little more than psychological understandings of inner personality traits such as ambition or predisposition to risk taking, and assumed to be accounted for in residuals.

Some scholars used generalized international surveys such as the European Social Survey and World Values Survey to explore migrant/non-migrant differentials in achievement-related motivational orientations (Polavieja et al., 2018). Alternatively, given the scarcity of high-quality representative data sources, others focused on data from countries of origin to explore differences between prospective migrants while still in origin and those who do not intend to migrate (Cebolla-Boado and Soysal Nuhoḡlu, 2017). On the whole, however, most studies simply refer ex-post to unobservable selectivity when accounting for unexplained individual variation.

In this paper, we attempt to overcome this limitation in the literature by empirically specifying unobserved selectivity among highly educated migrants, which has rarely been an empirical focus in migrant selectivity research. Despite the fact that international higher education students are now broadly regarded as integral to high-skilled migratory flows in global indicators (Hawthorne, 2008; OECD, 2017), the expansive literature on international education hardly dialogues with migration studies. Our paper explicitly links higher education and migration research fields, by using the *Bright Futures* dataset,^1^ a unique, large-scale dataset of about 8,000 Chinese international tertiary students and their native-born counterparts in China, the UK, and Germany. In the G20 area as a whole, half of all international students come from Asia, with China, followed by India and Korea, being the main contributors (OECD, 2017). Furthermore, Chinese students make up over 20% of international students in all OECD countries and constitute the largest and fastest-growing body of students from any single country. Although the US is the top destination for Chinese students, the UK attracts the highest numbers in Europe with 10% and Germany 3% as the third choice study destination in the region. To the best of our knowledge, this is the first ever source of systematically representative data to allow thorough research designs on a single flow of highly educated migrants with control groups in origin and destination. Our paper seeks to empirically identify unobserved selectivity in order to confirm whether selection, most often studied using observable indicators among economic and unskilled migrants, actually takes place due to unobservables among the most educated.

## Research Hypotheses: Agentic Individuality as the Source of Unobservable Selectivity?

The growing research on migration selectivity has largely ignored the specificities of differentiated groups of migrants such as humanitarian, unskilled and skilled, and international students. While there is an increasing amount of evidence confirming selectivity among unskilled labor migrants and migrant populations at the aggregate level as well as research pointing to selectivity on observable characteristics among international students (Brooks and Waters, 2011; Findlay et al., 2012; Gerhards and Hans, 2013), overall selectivity patterns among the skilled and educated migrants are not so well-understood. We start with the proposition that selectivity patterns among the educated are likely to differ from those of the general migrant population. A highly significant aspect of contemporary education is the emphasis it places on the increasingly standardized models of the agentic individual, with expanded notions of rights and capabilities, which defines proactive, independent, and goal-oriented individuals (Meyer and Jepperson, 2000). The current neoliberal contexts, with their focus on knowledge economies, anticipate such traits to impact achievement and success in education, labor markets, and overall life goals (Soysal Nuhoḡlu, 2012; Hasse and Krucken, 2013). Since the 1990s, the agentic individual model has been transmitted, not only to students but to broader society as well, through scientific theories and ideologies of education, creating uniform expectations and equipping individuals on a global level with such narratives of the self (Frank and Meyer, 2002; Soysal Nuhoḡlu and Wong, 2007, 2015; Lerch et al., 2017). Particularly in higher education, which is a highly transnationalized field, we observe a standardized conception of the student that centers around individual agency, ambition, competitiveness, and openness to new experiences. This conception cuts across higher education sectors the world over, affecting self-orientations and perceptions of not only those who migrate for their education but also those who stay. As tertiary education may well be playing the role of “equalizer” of aspirations, ambitions, and orientations (Karlson, 2018), selectivity among highly educated migrants might be overstated in the literature.

Accordingly, we suggest two alternative hypotheses:

1. Given the prior evidence of selectivity among the general migrant population, we expect Chinese students who migrate for their tertiary education to be positively selected on individuality traits when compared with those who stay in China.
2. Given that higher education students are heavily exposed to standardized models of the agentic individual, we expect no differences on expressed individuality characteristics between Chinese students who migrate and those who do not.

While the first hypothesis requires comparison between migrants in destination and non-migrants in origin, confirming the second necessitates multiple comparisons between migrants and those native-born in origin and destination and, inevitably, in more than one host society, since the argument is that tertiary education foments similar orientations among students on a global scale. The *Bright Futures Survey* helpfully includes Chinese students and native-born students in two European societies: the UK and Germany.

## Data and Methods

### Survey Data

The *Bright Futures Survey* (http://brightfutures-project.com/technical-report/) is a multi-country survey of students enrolled in tertiary education in China, Germany, and the UK. The questionnaire was carried out in Mandarin Chinese, English, and German with about 8,000 students in all three countries. The fieldwork was conducted in 2017 and 2018, using different sampling strategies in Europe and China. After thorough research into how Chinese international students are sorted across universities in their chosen destinations (Cebolla-Boado et al., 2017), a two-stage sampling logic was adopted in Germany and the UK. Universities were first stratified into groups according to ranking and number of Chinese students enrolled in each institution to ensure that students from different types of universities were appropriately proportionally represented. Within each university selected, random samples of undergraduate and master's students of Chinese and native backgrounds were obtained and individually invited to answer the questionnaire online. For China, the sample was stratified to cover different provinces in the north, south, and east of the country and take into account university prestige.

Table 1 describes the sample sizes for each of the analytic groups in this paper: international Chinese students in tertiary education in the UK and Germany, Chinese students studying in China, and two control groups of British and German students that we use in the main descriptive analysis. Note that the samples of British and German students are not representative of the universe of tertiary students in the UK and Germany but only of native-born students matriculated in universities in which Chinese students are also matriculated.

**Table 1:** Bright future survey sample sizes.

**Country**	**Sub-sample of students**	**Frequency**
UK	Chinese international	1,523
	British	1,730
Germany	Chinese international	814
	German	425
China	Chinese	3,427
	Total	7,919

*Bright Futures Survey*.

The British sample of Chinese students is fully representative of the universe of Chinese students in the UK and implements the sampling approach successfully, covering the entire universe of British higher education institutions and Chinese students across them. Similarly, the British sample is representative of native-borns enrolled in those higher education institutions in which Chinese in our sample are studying. The German sample of Chinese students did not cover the entire universe of Chinese students in Germany, and as such cannot be considered fully representative, and the sample of native Germans is smaller than that of Chinese. We found, however, that the differences between the UK and Germany are unremarkable in terms of our interests and the independent variables we use in the following analyses. In sum, our comparisons between China and the UK are based on representative samples, while we present results using the German sample to increase the robustness of our results. Note that replicating the analyses we present here without the German sample provides identical substantive results.

### Variables Used in the Analyses and Methods

Socioeconomic background is commonly considered when studying observable migrant selectivity. We use the father's occupation (e.g., a dummy combining professional, technical, and high-level administration vs. the rest) and education (whether the father is a university graduate) as socioeconomic background variables. However, our main interest in this paper is to delve into a less commonly studied aspect of migrant selectivity, namely, selection on unobserved characteristics. There is not a large research tradition investigating differences between migrants and non-migrants in terms of unobserved characteristics. Following our argumentation, we use four different aspects that look at agentic individuals as embedded in broader educational frameworks. *Bright Futures Survey* included four questions asking students if someone who “thinks up new ideas” (creative), someone who “makes their own decisions” (independent minded), someone who “looks for adventures and taking risks” (risk-taker), and someone who “values being successful” (achievement oriented) was “not at all like s/he,” “somewhat like s/he,” “neither like s/he nor unlike s/he,” “somewhat like s/he,” or “very much like s/he.” It is important to note that we understand the individual characteristics represented in each question as self-perceptions and representations, much shaped by broader educational scripts and frameworks, rather than inner and habitual personality traits as suggested in the psychological literature. Given the strong socialization role of education and broader societal expectations of self-development, however, it is possible that the gap between self-perceptions and habitual personality traits may well be narrow among the population we are focusing on.

The survey questions above are the main variables used in our empirical sections focusing on unobservable selectivity. In our analysis, with answers to each question recoded into dummies, taking the value of 1 for the first two categories of answers (“somewhat” and “very much like”) and 0 for the remaining three, we estimated separate models. In this exploratory stage, linear probability models and logistic regressions were estimated to capture the average answer given by students in all five of our analytic categories (Chinese in China, the UK, and Germany, and both groups of European students). The Appendix includes four sets of overlapping histograms (Figures A.1–A.4) in which the distribution of each of these variables (individual traits) is compared across groups (Chinese in China and the UK, Chinese in China and Germany, Chinese in the UK and British, and Chinese in Germany and Germans).

As a second step, we merged all four variables into a synthetic index of agentic individual characteristics (results of the principal component analysis are presented in the Appendix, Table A.1. Figure A.5, also in the Appendix, plots the distribution of the resulting index). This continuous variable is used as the explicandum of a doubly robust treatment effect model (Linden et al., 2016) using inverse probability weighting with regression adjustment (IPRWA), in which the treatment takes the value of 1 if the respondent is a Chinese student who migrated to Germany or the UK and 0 for Chinese students in China. The advantage of estimating IPRWA treatment effects is that one can first model selection into treatment (in our case migration status) and then match comparisons from treated and control groups to measure the average treatment effect that, in our analyses, corresponds to the net differences in our index of agentic individual characteristics for migrants and non-migrants. It is possible that students from highly educated families are more likely to be exposed to agentic individual ideals (as these ideals spread through education) and are thus more likely to migrate. In consequence, we expect family educational background to play an intermediary role in positive selection on observed characteristics of those Chinese students who go abroad. Furthermore, given that studying abroad brings financial costs, we expect that there is also selectivity on the basis of parental occupational background. In our treatment effects estimation, we use a probit regression to model selection into migration using father's education (1: university degree; 0: other) and father's occupation (1: professional, technical and high-level administration; 0: other). We also use a recalled proxy of ranking in high school (1: if the student reports being in the 5th percentile; 0 otherwise). These three variables, measuring selection on basic observables (i.e., social background and previous performance), which the literature argues to be relevant for educational migration, help us discount from the association between migration and individual characteristics. Our model also controls for the propensity to migrate among Chinese students in rural or urban settings in China (1: rural). Finally, note that our multivariate model further controls for student gender (1: female). The Appendix includes a table (Table A.2) reporting the basic description of all variables included in this analysis.

## Findings

### Descriptive Results

**Figure 1:**
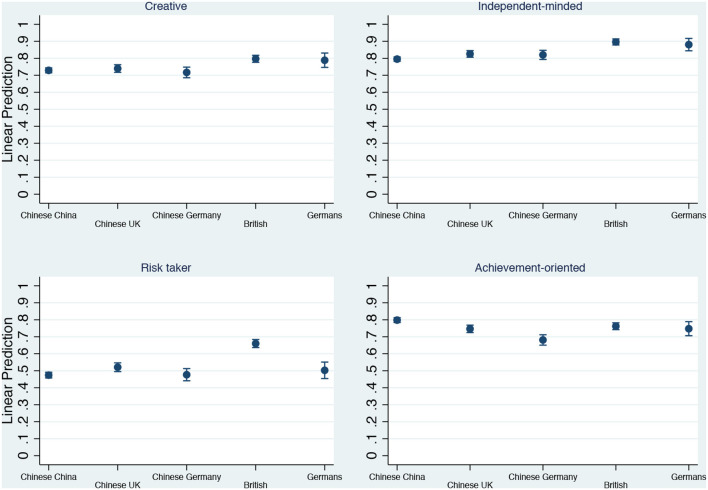
Differences in selected individual characteristic across student groups. Our elaboration from *Bright Futures Survey*. Estimates obtained from models in Table A.3 in the Appendix. Estimates and 95% confidence intervals.

The first empirical results correspond to the differentiated effect of student groups on individual characteristics. This is done using unconditional linear probability models (LPM)^2^. Figure 1 (obtained from models shown in Table A.3 in the Appendix) summarizes these results looking at average responses given across groups. Throughout the panels, it can be seen that differences across groups of students are, if anything, modest, not to say non-existent. The test for selectivity requires comparison of outcomes across Chinese students in the countries included in the analysis: China, Germany, and the UK. Doing so reveals that there are no major differences across respondents from this national origin by country of survey. While there are some signs of statistically significant difference between Chinese across groups, these are of a small substantive importance and do not consistently go in the direction the selectivity hypothesis would suggest. Compared to their national counterparts in origin, Chinese in the UK are 3 and 5% more inclined to identify themselves as independent minded and risk-taker, respectively; however, they also report that they are 5% less achievement oriented (in the case of Chinese in Germany, 12%). On the other hand, Chinese in the UK and Germany are equally inclined to say that they are as creative as Chinese in China, and we see no differences between Chinese in Germany and Chinese in China in the likelihood of reporting being independent minded and risk-taker.

Overall, our results fit better the agentic individual hypothesis. The hypothesis suggests that the distribution of individual characteristics across student nationalities and countries of survey should be similar, since transnationalization implies a diffusion process whereby the model becomes taken-for-granted independently of national contexts (Soysal Nuhoḡlu, 2015). Thus, we expect respondents' self-definitions to converge. This is indeed what we find as graphically summarized in Figure 1, obtained from unconditional LPM. While Europeans on the whole score slightly above Chinese respondents, the differences are small in size. In the first panel, around 80% of Europeans indicate that someone creative is “very much” or “somewhat” like them, while this figure is around 70–75% among Chinese students in all three countries. Similarly, a small gap between Chinese and Europeans also appears in the second panel; around 80–85% of Chinese and 90% of Europeans recognize themselves as independent minded. The third panel, where being a risk-taker is the object of interest, is the only one in which we find some differences between the student groups. While 45–50% of Chinese students in Europe and China fall into the risk-taker profile, a similar percentage to that displayed by German students, a higher percentage of British students, 65%, identify as such. While this is a considerable gap, the broad similarities between Chinese and German students are in line with the predictions of the agentic individual hypothesis. Finally, the fourth panel fully fits with the expectation that all students score similarly across national origins and countries of survey−70–80% of students in all categories claim to be achievement oriented. In other words, this preliminary and unconditional analysis suggests that there are no clear grounds for arguing that there is selection on unobservables among students in tertiary education who migrated to other countries to pursue their degrees. It is rather the opposite; a remarkable homogeneity in how students perceive themselves dominates, which suggests that conceptions of the self are rather transnationalized among individuals who have already made it into tertiary education. This finding points to the increasingly standardized nature of university students across higher education contexts.

The standardization of the agentic individual model among university students stands clear when we further disaggregate the analysis by gender. The Appendix includes a replication of these plots, splitting the sample by respondents' gender in order to discard the possibility of agentic individual characteristics being patterned differently across different groups of students in terms of gender (Figure A.6). The plots confirm that, unlike predictions of selectivity hypothesis, we do not find any systematic differences neither between Chinese students in different countries nor between Chinese and native-born students in European destinations in regard to agentic characteristics. Compellingly, male and female respondents do not differ in the importance they attribute to these characteristics when describing themselves.

**Figure 2:**
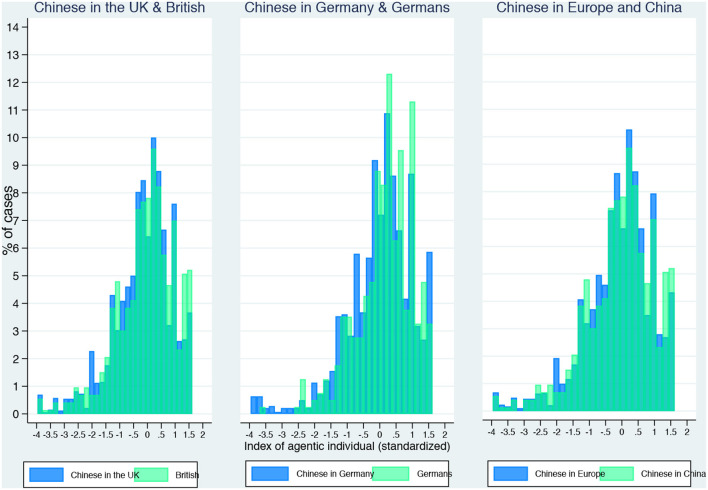
Distribution of the synthetic score of agentic individuality by analytic groups. *Bright Futures Survey*.

After merging these different components of the agentic individuality into a synthetic dimension using principal component analysis (see Table A.1 in the Appendix), we confirm the remarkable similarities in the distributions of this factor across our analytical categories in Figure 2. This provides a more intuitive visual confirmation of our second working hypothesis. In the multivariate analysis that follows comparing Chinese students in China and Europe, we use this factor as our dependent variable of interest.

### Multivariate Analyses

While our preliminary and unconditional conclusions already suggest that higher education leaves no room for migrant selectivity, it is necessary to discard systematic composition effects associated with migration in order to attain a more conclusive view on whether migration does indeed signify positive selection of individuals on unobservables. Isolating the effect of migration on any specific individual characteristic or behavior ideally requires longitudinal multi-sited data that link countries of origin and destination in order to identify the distinctive features of migrants and native-borns in origin and host societies. These distinctive features could be the result of the fact that individuals who migrate, even before they make the move, can be systematically different to those who prefer to stay. Alternatively, the systematic differences between migrants and native-borns in origin may result from the very nature of the formers' experience of migration. Detecting which of these two possibilities applies in our case is a complex task particularly with the kind of observational, cross-sectional data that we use. Nonetheless, treatment effects and other quasi-experimental research methods allow us to model differences between treatment and control groups (i.e., migrants vs. non-migrants, in our case), controlling for a list of relevant regressors and selection into treatment. Specifically, selection into treatment (migrating being the treatment and not doing so being the control) is essential in order to disentangle whether differences by treatment status are due to selection or to experiencing migration. Only after modeling selection can we adjust the regression using other covariates and thus credibly confirm whether being a migrant implies any difference in the individual characteristics with which we are concerned when compared with non-migrants. In the lack of longitudinal data, this modeling approach represents the best alternative in estimating our effect net of selection into migration, since it circumvents the most important limitation inherent to cross-sectional observational data, that is, the non-random allocation of migrant status among migrants and non-migrants. Furthermore, treatment effects with inverse probability weighting is a double robust estimation method, which implies that the estimators are unbiased if at least one of the equations is correctly specified (Funk et al., 2011).

Table 2 shows our results. Let us first focus on the probit regression predicting the treatment status (migration). Chinese students in Europe and China were included in the analytic sample. As the literature on international education suggests a positive selection of international students by social background, we used two relevant predictors modeling this family condition: father's occupation in professional, technical, and high-level administration, and father's highest level of education being a tertiary degree. Research focusing on brain drain in migration suggests international students being positively selected on school performance. Thus, in our treatment equation, we included high school results (being in the 5th percentile of class in high school). We also control for whether the student comes from a rural or an urban setting in China. Our probit equation confirms that there is positive selection into international education by father's education and occupation, although these estimates are far from implying that all international students are from privileged social origins (having a father with a university degree or a father in the highest occupational social class increases the likelihood of being a migrant around 50%). Similarly, having a successful high school performance merely increases this probability by 10%. Students from rural settings in China are less likely to engage in educational migration.

**Table 2:** IPRWA treatment effect on the agentic individual score Chinese in Europe (T) and Chinese in China (C).

Average treatment effect		0.081
		(0.048)
Population means		−0.12^*^
		(0.023)
Regression adjustment: control	Father's occupation is professional and technical or high-level administration	0.014
		(0.061)
	Father has university education	0.14
		(0.071)
	Student is female	−0.21^*^
		(0.045)
	Constant	−0.037
		(0.035)
Regression adjustment: treatment	Father's occupation is professional and technical or high-level administration	0.0026
		(0.075)
	Father has university education	−0.013
		(0.065)
	Student is female	0.045
		(0.088)
	Constant	−0.062
		(0.086)
Selection into treatment	Father's occupation is professional and technical or high-level administration	0.55^*^
		(0.052)
	Father has university education	0.52^*^
		(0.055)
	Student is 5th percentile of the class in high school	0.096^*^
		(0.046)
	Rural setting in China	−1.07^*^
		(0.060)
	Constant	−0.54^*^
		(0.048)
*N*		4,165

*Our elaboration from Bright Futures Survey*.

Standard errors in parentheses;

* *p < 0.05*.

Our model estimates the “average treatment effect” (ATE) associated with being in the treated group compared to the control, net of selection and adjusted by a number of controls including parental occupation, parental education, and student gender. The results from this more demanding approach to estimating effects using observational data show that there are no grounds for claiming that educational migrants are positively selected on unobserved characteristics. The treatment group (Chinese migrants) scored on average 0.08 more in the synthetic score of agentic individuality than the control (Chinese non-migrants); however, this effect is not statistically significant. In other words, there is no sign of migrant selection in agentic individuality, when the empirical focus is on highly educated populations.

### Robustness Checks

Our synthetic factor of agentic individual personality is consistent among analytic groups. We have re-estimated our principal component analyses for each of them separately with identical results to those here reported. We have also re-estimated our analysis using a different sample of Chinese educational migrants in Japan with very similar results to those discussed in this paper. Our multivariate results are stable controlling for other potentially relevant individual characteristics such as age, year of education (1st, 2nd, or 3rd years), and level of studies (master's vs. undergraduate degrees).

## Discussion of the Results and Implications

Studying migration selectivity has become a priority topic for current migration scholarship. Our paper overcomes two important limitations in this research agenda. Firstly, selectivity research up until now concentrated on the entire stocks of migrants in destination countries, where low skilled economic migrants prevail, in the process overlooking whether highly educated and skilled migrants are any different. Our paper, to the best of our knowledge, is the first to produce a systematic documentation of patterns of selectivity for a specific flow of highly skilled, international students combining data from origin and destination. Secondly, the selectivity research agenda prioritized observable socioeconomic background variables. Ready availability of such indicators in many standard surveys explains this preference. However, the most intriguing regularities in research into integration in which migrants appear as the advantaged population (such as the “paradox of immigrant optimism”) correspond to selection on ambition and similar individual traits that most often remain unobserved and are simply mentioned as ex post attributes to unexplained residual variation. Consequently, more often than not, research into selectivity downgrades the role of unobserved characteristics.

In this paper, we set ourselves the task of explicitly studying unobservable selectivity. By taking into consideration the broader institutional contexts that frame individuals' self-expressions of worth and traits, we were able to theorize about and empirically specify unobserved individual selectivity, beyond assumed personality attributions assigned to unexplained residuals. We considered how four specific individual characteristics—being creative, independent minded, a risk-taker, and achievement oriented—are distributed among migrants and non-migrants from different origins. We acknowledge that this is not a comprehensive list of characteristics that might be relevant to research into migrant selection on unobservables. However, these individual characteristics, with attributed agency, are of particular importance because of the place they have in broader institutional frameworks that privilege knowledge economy, which is regarded as the driver of the current migration flows of the most skilled and educated.

Our research into the international migration of Chinese tertiary students shows that selection among these educational migrants occurs, to a certain extent, due to observable characteristics, such as social background (parental occupation and education) and prior academic performance, yet these are small effects, suggesting that educational migration is a rather heterogeneous migration flow. For our central concern in this paper, however, we document no selection on the basis of what is often attributed to the unobserved. Our evidence shows that Chinese university students who migrated for their studies are equally likely to see value in individual characteristics such as being creative, independent minded, a risk-taker, or achievement oriented as their peers who did not make the move abroad. Furthermore, they are also similar to students matriculated in British and German universities who were natively born. In other words, there is a remarkable convergence in how youth define themselves across countries and migrant status, which leaves no room for claiming that our target population is positively selected.

We offer explanations for this empirical regularity by highlighting the highly transnationalized education systems across the globe that play a predominant role in standardizing the idea of agentic individuals and their aspirations as worthy, not only for individual but also national and global futures. This idea has become embedded in a variety of institutions beyond education in the liberal and neoliberal context of the last 50 years. It has been promoted by international organizations (such as the cultural conventions of UNESCO and the Council of Europe) and is found in human rights frameworks, global art platforms, organizational managerial ideologies, and market-driven national and international institutions (in the health, IT, and finance sectors) (Hall and Lamont, 2009; Soysal Nuhoḡlu, 2012; Bromley and Meyer, 2015). Future research might consider selectivity in migration flows materializing in connection with these different social domains.

It might be argued that our findings in this paper are driven by the empirical choice of a certain migrant group, that is, Chinese international students. China experienced a late expansion of its tertiary education after the country's opening up in the 1980s. China's highly stratified university system (through the centralized arrangement of university admissions) and the rapid expansion of its middle classes created massive internal competition for places in prestigious universities. This, it might be argued, helps to explain the outmigration of higher education students with a homogeneous profile. However, our findings confirm a high level of convergence between Chinese students and their British and Germans counterparts, which cannot be explained by such internal dynamics. Future research into other higher education contexts in which internal competition for highly ranked universities is not so fierce, as is the case in many African and Latin-American countries, could well make evident the robustness of our findings and explanations.

Finally, we believe that our paper convinces due to the availability of data, which makes it possible to observe educational migration selectivity across two destinations, the UK and Germany. These two destinations have different positions in relation to highly skilled migration flows, the UK being the only European country to have had significant success in the so-called “Global Race for Talent,” in which Germany lags behind. Evidence of similar patterns of selectivity in educational migration to these different destinations is further support for the argument we put forward in this paper. Given the increasing proportion of the highly skilled and educated in contemporary migration flows, with increasingly heterogeneous destinations, empirical research expanding beyond North American and European contexts could be fruitful for future study.

## Data Availability Statement

The datasets for this study will not be made publicly available because the dataset is currently under embargo, and will be publicly available in 2021. We will however provide stata syntax files on request.

## Ethics Statement

This study was carried out in accordance with the recommendations of the Statement on Safeguarding Good Scientific Practice by the Ethics Committee of University of Essex with written informed consent from all subjects. All subjects gave written informed consent in accordance with the Declaration of Helsinki. The protocol was approved by the University of Essex Ethics Committee.

## Author Contributions

All authors listed have made a substantial, direct and intellectual contribution to the work, and approved it for publication.

### Conflict of Interest

The authors declare that the research was conducted in the absence of any commercial or financial relationships that could be construed as a potential conflict of interest.

## Footnotes

^1^“Bright Futures”: A Comparative Study of Internal and International Mobility of Chinese Higher Education Students. Principal Investigator: Yasemin Soysal Nuhoḡlu (University of Essex), Co-Investigators: Héctor Cebolla-Boado (UNED), Thomas Faist (University of Bielefeld), Jingming Liu (Tsinghua University), and Sophia Woodman (University of Edinburgh). The project was funded jointly by the Economic and Social Research Council, Deutsche Forschungsgemeinschaft, and the National Natural Science Foundation of China.

^2^Logistic regression with average marginal effects did not produce any substantive changes in the results discussed here.
