# Supplementary material for: Observing the Unobservable: Migrant Selectivity and Agentic Individuality Among Higher Education Students in China and Europe
Source: Front Sociol. 2020 Mar 13;5:9. doi: 10.3389/fsoc.2020.00009 (PMC8022453; doi:10.3389/fsoc.2020.00009)
Supplement: Supplementary file 1 [file Data_Sheet_1.pdf]

APPENDIX

Figure A.1. Distribution of components of the agentic individuality factor by analytic groups: creative.

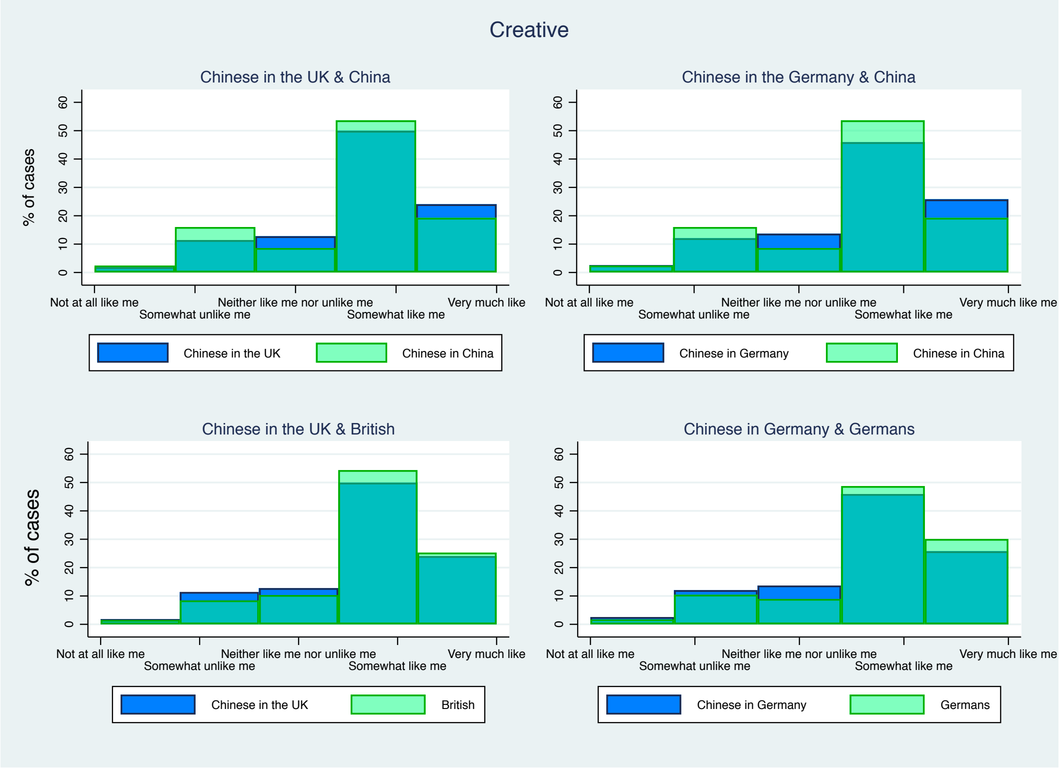

Source: *Bright Futures Survey*.

Figure A.2. Distribution of components of the agentic individuality factor by analytic groups: independent minded.

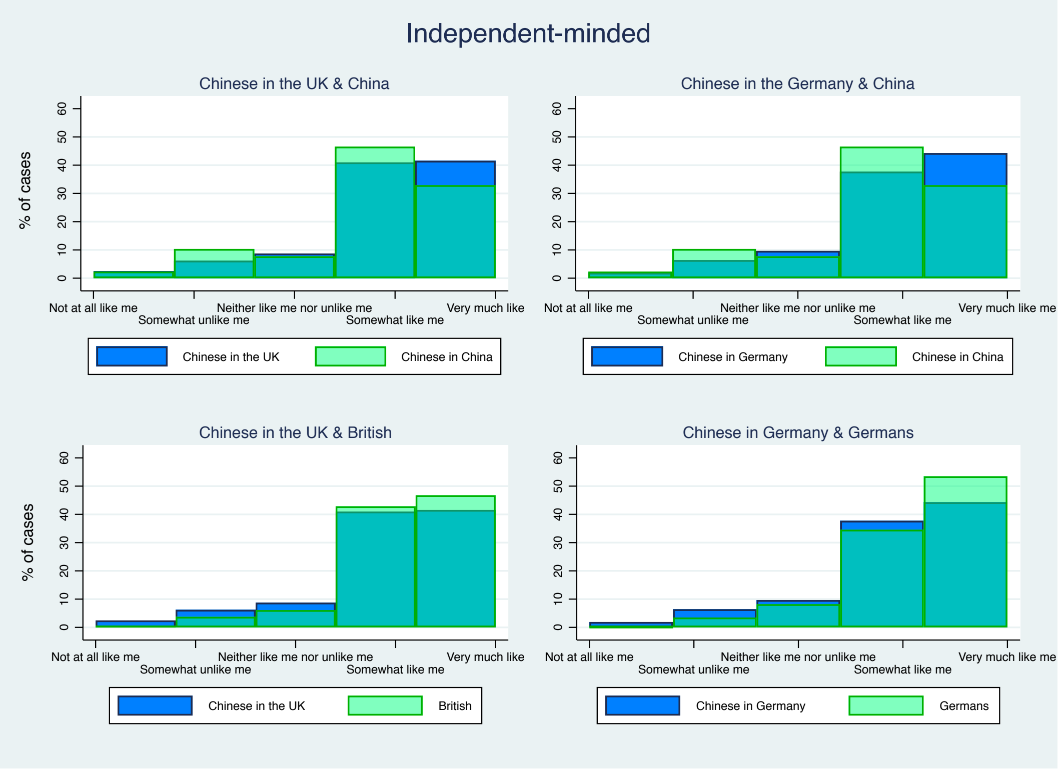

Source: *Bright Futures Survey*.

Figure A.3. Distribution of components of the agentic individuality factor by analytic groups: risk-taker.

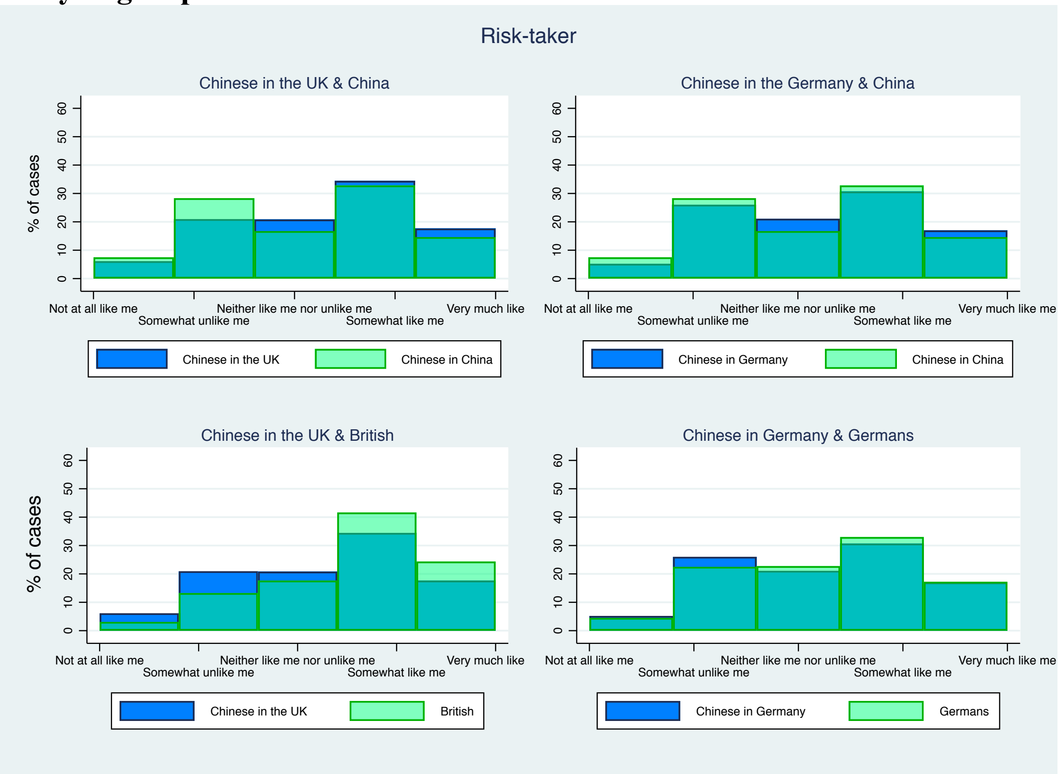

Source: *Bright Futures Survey*.

Figure A.4. Distribution of components of the agentic individuality factor by analytic groups: achievement oriented.

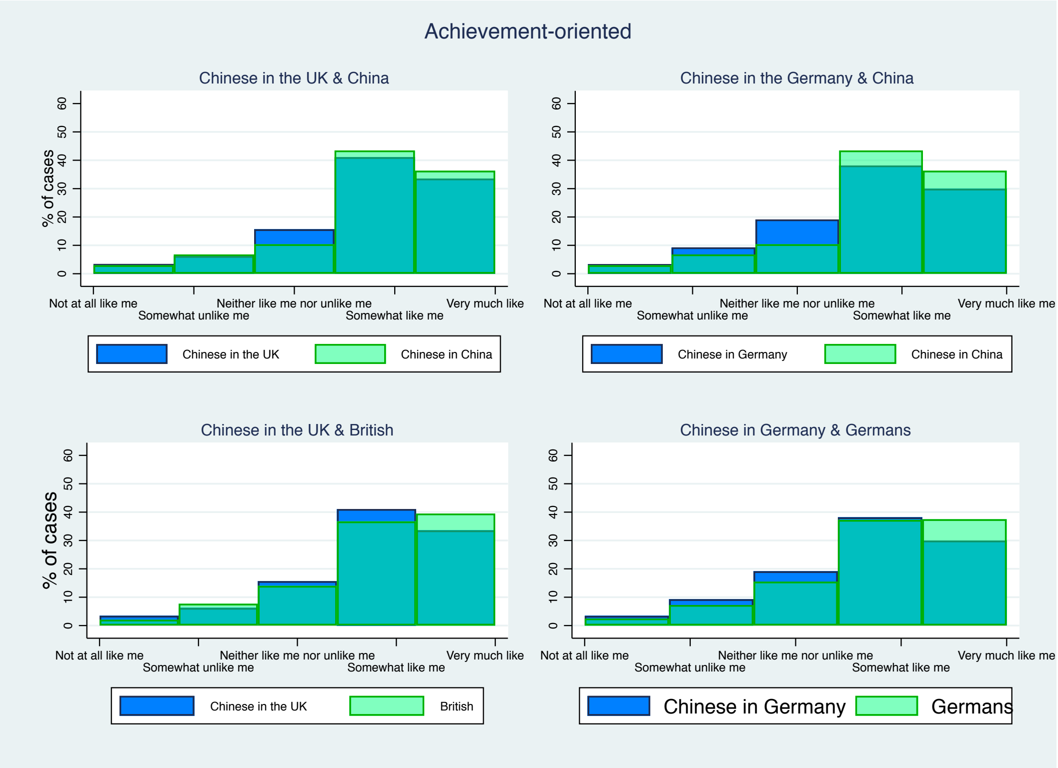

Source: *Bright Futures Survey*.

Table A.1. Principal component analysis

Selected component	Eigenvalue	Proportion
Agentic individual	1.90	0.47

Eigenvectors	Agentic individuality index
Creative	0.5528
Independent-minded	0.5513
Risk-taking	0.4884
Achievement oriented	0.3898

Figure A.5. Kernel distribution of the agentic individual index.

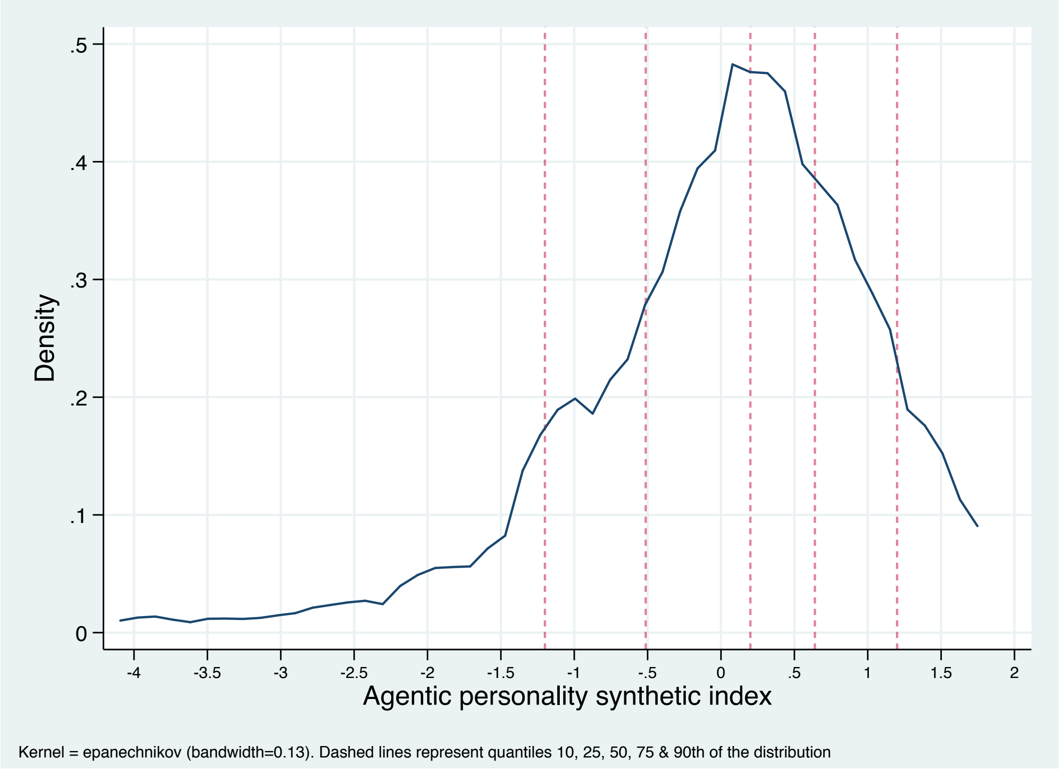

Source: Our elaboration from *Bright Futures Survey*.

Table A.2. Description of variables used in the empirical analyses

	Variable	Mean	Std. Dev.	Min	Max
Agentic individual characteristics		−0.13	1.4	−5.5	2.2
Father's occupation	Professional and technical or high-level administration	0.46	0.50	0	1
Father's education	University	0.30	0.50	0	1
Student female		0.59	0.49	0	1
Student 5th percentile of class in high school		0.62	0.48	0	1
Student rural setting in China					

Source: Our elaboration from *Bright Futures Survey*.

Table A.3. Linear Probability Models on individual characteristics by sample

	**(1)**<br>Creative	**(2)**<br>Independent-minded	**(3)**<br>Risk-taker	**(4)**<br>Achievement oriented
Chinese in UK	0.011	0.030*	0.047*	−0.052*
(ref. Chinese in China)	(0.014)	(0.012)	(0.016)	(0.013)
Chinese in Germany	−0.012	0.025	0.0024	−0.12*
	(0.018)	(0.015)	(0.020)	(0.017)
British	0.068*	0.10*	0.19*	−0.036*
	(0.013)	(0.011)	(0.015)	(0.013)
Germans	0.059*	0.085*	0.028	−0.051*
	(0.023)	(0.020)	(0.026)	(0.022)
Constant	0.73*	0.80*	0.47*	0.80*
	(0.008)	(0.007)	(0.009)	(0.007)
F	8.91	22.4	42.2	13.1
N	7534	7534	7534	7534
R^2	0.0047	0.012	0.022	0.0069

Source: Our elaboration from *Bright Futures Survey*.
Standard errors in parentheses; $*p < 0.05$.

Figure A.6. Differences in selected individual characteristics across student groups by gender.

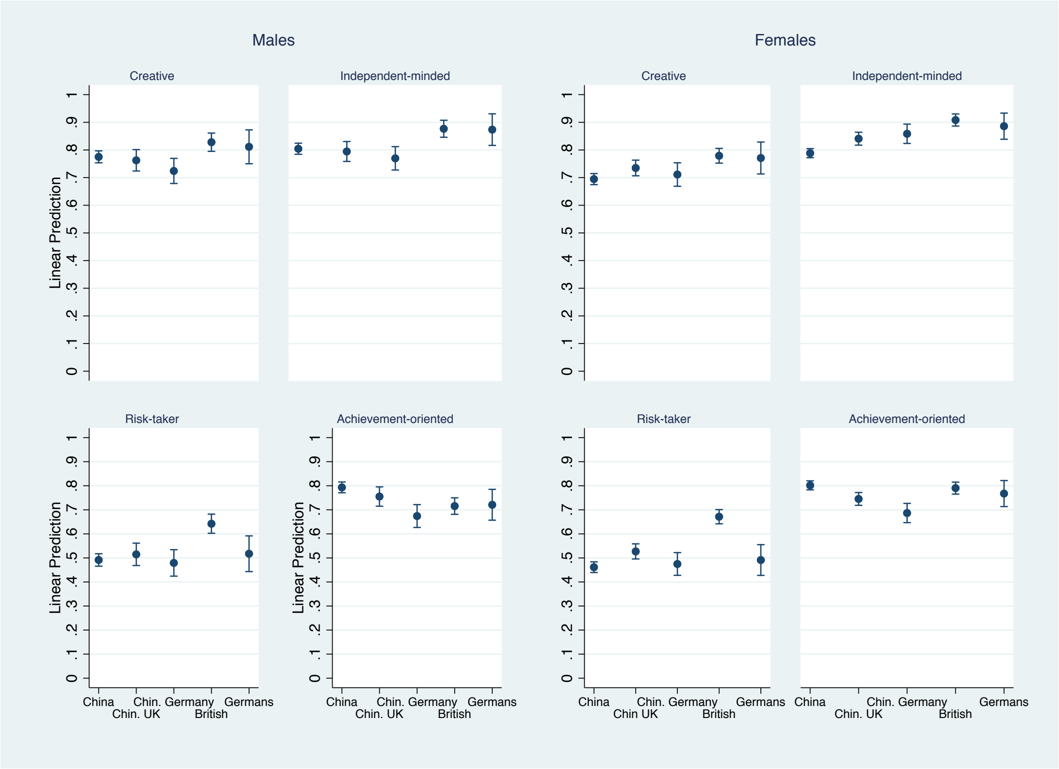

Source: Our elaboration from *Bright Futures Survey*. Estimated from models not shown in the paper but available upon request. Estimates and 95% confidence intervals.
